# Fecal microbiota transplantation promotes gut microbiome recovery in pediatric hematopoietic stem cell transplant recipients

**DOI:** 10.3389/frmbi.2026.1849762

**Published:** 2026-06-05

**Authors:** María Florencia Fernandez, Abigail Stricker, Adriana Bottero, Laura Busquet, Carlos Waldbaum, Fabiana López Mingorance, Raúl Martinez Patetta, Ignacio Toer, Ana Juliá, Andrea Mangano

**Affiliations:** 1 Department of Microbiology, Hospital de Pediatría “Prof. Dr. Juan P. Garrahan”, Buenos Aires, Argentina; 2 Department of Gastroenterology, Hospital de Pediatría “Prof. Dr. Juan P. Garrahan”, Buenos Aires, Argentina; 3 Division of Gastroenterology, Hospital de Clínicas “José de San Martín”, Buenos Aires, Argentina; 4 Bone Marrow Transplant Unit, Hospital de Pediatría “Prof. Dr. Juan P. Garrahan”, Buenos Aires, Argentina

**Keywords:** 16S rRNA sequencing, acute graft-versus-host disease, dysbiosis, fecal microbiota transplantation, gut microbiome, microbiome diversity, pediatric hematopoietic stem cell transplantation

## Abstract

**Introduction:**

Hematopoietic stem cell transplantation (HSCT) profoundly disrupts the gut microbiome and may contribute to adverse post-transplant outcomes. Fecal microbiota transplantation (FMT) has emerged as a strategy to restore microbial diversity; however, data in pediatric HSCT recipients remain limited.

**Methods:**

We conducted a longitudinal analysis of 17 pediatric HSCT recipients who received FMT. Fecal samples were collected before FMT and at days 7, 14, and 30 after treatment. Gut microbiome composition was analyzed using 16S rRNA gene sequencing.

**Results:**

Baseline samples showed reduced microbial diversity and a dysbiotic microbial profile. Following FMT, microbial diversity increased progressively, with recovery evident from day 7 and stabilization by day 30. Taxonomic analyses demonstrated depletion of dysbiosis-associated genera and enrichment of beneficial short-chain fatty acid–producing taxa, including *Faecalibacterium*, *Blautia*, *Subdoligranulum*, and *Akkermansia*. Distinct microbial configurations were observed according to gastrointestinal involvement by acute graft-versus-host disease.

**Conclusions:**

FMT was associated with progressive restoration of gut microbiome diversity and structure in pediatric HSCT recipients, supporting its potential role as a microbiota-based strategy to promote ecological recovery after HSCT.

## Introduction

The gut microbiome has emerged as a central determinant of host metabolism, mucosal integrity, and immune regulation. Beyond its taxonomic diversity, this complex ecosystem contributes to nutrient absorption, epithelial barrier maintenance, and modulation of inflammatory and immune pathways (Schirmer et al., 2018; Zheng et al., 2020). Disruption of this equilibrium, commonly referred to as intestinal dysbiosis, has been associated with the depletion of beneficial commensals and the expansion of opportunistic taxa linked to infection, mucosal injury, and adverse clinical outcomes (Lloyd-Price et al., 2017; Shen et al., 2025).

In pediatric populations, the intestinal microbiome undergoes rapid and dynamic maturation during early life, evolving from a low-diversity, aerobe-dominated community to a stable anaerobic ecosystem resembling that of adults (Yatsunenko et al., 2012; Mohammadkhah et al., 2018). This developmental plasticity makes the pediatric gut particularly susceptible to environmental and iatrogenic perturbations, including antibiotic exposure, chemotherapy, and hospitalization (Tamburini et al., 2016; Yassour et al., 2016).

Hematopoietic stem cell transplantation (HSCT) represents one of the most disruptive clinical contexts for the gut microbiome. Conditioning regimens, broad-spectrum antibiotics, mucosal injury, and immunosuppression induce profound alterations in intestinal homeostasis and lead to marked dysbiosis (Biagi et al., 2015; Ugrayová et al., 2022). This imbalance has been strongly associated with an increased risk of bloodstream infections, colonization by multidrug-resistant organisms, and acute graft-versus-host disease (aGvHD), a major cause of transplant-related mortality affecting approximately 50–60% of allogeneic HSCT recipients (Peled et al., 2020; Chang et al., 2021; Hong et al., 2021). Early recognition of systemic infection in HSCT recipients remains challenging because inflammatory responses may be altered by neutropenia and immunosuppression. Recent evaluations of immunological and biochemical biomarkers for sepsis severity suggest that microbiome-related risk factors may be considered alongside inflammatory biomarkers when monitoring vulnerable post-transplant patients (Alshammary et al., 2025). Low microbial diversity and Enterococcus dominance before engraftment have been linked to a higher incidence and severity of aGvHD (Taur et al., 2014; Stein-Thoeringer et al., 2019). Moreover, reduced abundance of obligate anaerobic taxa, particularly members of the order *Clostridiales*, including *Faecalibacterium* and *Ruminococcus*, has been reported (Malard et al., 2018; Henig et al., 2021). Consequently, gut microbiome dysbiosis is associated with increased susceptibility to systemic bacterial infections, promotion of GvHD development, and higher risk of relapse (Biagi et al., 2019; Devaux et al., 2020).

Given the strong relationship between intestinal dysbiosis and transplant outcomes, microbiota-targeted therapeutic strategies have been proposed to restore microbial integrity and mitigate post-transplant complications, including fecal microbiota transplantation (FMT). FMT has demonstrated efficacy and safety in restoring microbial balance in recurrent *Clostridioides difficile* infection, and recent studies have explored its role in HSCT recipients to promote microbiome recovery and modulate immune-mediated complications (Shogbesan et al., 2018; Biernat et al., 2020; Henig et al., 2021). However, data in pediatric HSCT populations remain scarce, and the longitudinal dynamics of microbiome reconstitution following FMT are still poorly understood. Therefore, the objective of this study was to evaluate the longitudinal dynamics of gut microbiome composition in pediatric HSCT recipients undergoing FMT, focusing on temporal changes in microbial structure and the recovery of key bacterial taxa associated with ecosystem restoration.

## Materials and methods

### Sample collection and storage

Fresh fecal samples from pediatric hematopoietic stem cell transplantation (HSCT) recipients followed at Hospital de Pediatría “Prof. Dr. Juan P. Garrahan” between 2018 and 2022 were collected in sterile stool containers. Donor material was obtained from the Fecal Microbiota Bank of Hospital de Clínicas (Universidad de Buenos Aires, Buenos Aires, Argentina). Healthy stool donors were selected according to standardized institutional screening protocols, including negative bacteriological, virological, and parasitological testing prior to donation. Donor preparations consisted of pre-filtered fecal suspensions stored at −80 °C and transported under frozen conditions to preserve bacterial viability and community structure. Each recipient received FMT material derived from a single donor, with no pooling of samples.

Recipient samples were collected at baseline (pre-FMT), defined as the time point following recovery from the post-transplant neutropenic phase and immediately prior to FMT. Patients subsequently received two consecutive FMT doses administered 24 hours apart via a nasoduodenal tube. Follow-up samples were collected at days +7, +14, and +30 after FMT. Fresh samples were immediately processed for nucleic acid extraction to preserve microbial integrity.

The study was approved by the Institutional Review Board (Comité Revisor y de Ética en la Investigación, Hospital de Pediatría “Prof. Dr. Juan P. Garrahan”, Protocol No. 1452), and written informed consent was obtained from parents or legal guardians.

### Nucleic acid extraction

Total nucleic acids were extracted from 0.5 g of fecal material using the DNA/RNA All Viral Power Kit (Qiagen, Hilden, Germany), according to the manufacturer’s instructions. DNA concentration and purity were assessed using a NanoDrop spectrophotometer (Thermo Fisher Scientific, Waltham, MA, USA). Extracted DNA was stored at −20 °C until further processing.

### 16S rRNA gene amplification and sequencing

The V3–V4 hypervariable regions of the bacterial 16S rRNA gene were amplified using universal primers 341F (5′-CCTACGGGNGGCWGCAG-3′) and 805R (5′-GACTACHVGGGTATCTAATCC-3′) (Klindworth et al., 2013). PCR products were purified using AMPure XP magnetic beads (Beckman Coulter, Brea, CA, USA). Library preparation followed the Illumina 16S Metagenomic Sequencing Library Preparation protocol, and sequencing was performed on the Illumina MiSeq platform using paired-end reads (2 × 300 bp).

Negative controls were included during DNA extraction and PCR amplification to monitor contamination from reagents and extraction kits. These controls were processed and sequenced in parallel with biological samples. Additionally, a defined bacterial community standard (ZymoBIOMICS Microbial Community Standard, Zymo Research, Irvine, CA, USA) was included as a positive control to assess sequencing accuracy.

### Bioinformatic and statistical analysis

Raw sequencing data were processed using the nf-core/ampliseq v2.12.0 pipeline (Straub et al., 2020). Sequence denoising and amplicon sequence variant (ASV) inference were performed using DADA2 v1.30.0 (Callahan et al., 2016). Reads were quality filtered using a minimum Phred score of 24 and a maximum expected error of 2 per read, and sequences shorter than 50 bp were discarded. Taxonomic assignment was performed using the SILVA 138.1 database (Quast et al., 2013). Data were imported into R v4.4.3 and analyzed using the phyloseq package (McMurdie and Holmes, 2013). Sequencing depth was assessed using rarefaction curves for Observed richness, Shannon, and inverse Simpson indices, indicating adequate sampling depth. Rarefaction was used only for diagnostic purposes; all analyses were conducted on non-rarefied data. Alpha diversity was calculated using Observed richness, Shannon, and inverse Simpson indices. Pairwise longitudinal comparisons were assessed using the Wilcoxon signed-rank test. In addition, longitudinal changes in alpha diversity over time were evaluated using linear mixed-effects models with time modeled as a continuous variable and patient identity included as a random effect. For taxonomic analyses, ASVs were agglomerated at the genus level using tax_glom(). Relative abundances were calculated, and the top 20 genera per sample type were retained and renormalized to sum to 100%. Compositional changes were visualized using alluvial plots. Samples were stratified according to the pattern of aGvHD organ involvement (absence or isolated cutaneous involvement versus gastrointestinal involvement), and mean genus-level abundances were visualized using heatmaps. Microbial association networks were inferred using the NetCoMi package (Peschel et al., 2021), following the approach described by Rashidi et al. (2023). Correlations were estimated using the SparCC algorithm (Friedman and Alm, 2012), with zero replacement and centered log-ratio transformation. Only correlations with absolute values ≥ 0.3 were retained. The Jaccard index was calculated as the number of shared edges divided by the total number of unique edges across both networks, with values ranging from 0, indicating no shared edges, to 1, indicating complete edge overlap. To assess whether observed edge overlap was lower than expected by chance, sample labels were randomly permuted 999 times while preserving group sizes, networks were reconstructed using the same NetCoMi/SparCC workflow, and empirical p-values were calculated as the probability of obtaining a permuted Jaccard index equal to or lower than the observed value. P-values were adjusted for multiple comparisons using the Benjamini–Hochberg procedure.

### Use of large language models

A large language model (ChatGPT, OpenAI, GPT-5.3) was used exclusively for language editing and improvement of clarity in the manuscript. The model was not used for data analysis, data interpretation, or generation of scientific content.

## Results

The cohort comprised 17 pediatric patients, with a median age at HSCT of 8.2 years (range: 0.7–17.0), of whom 64.7% were male. Underlying diagnoses included acute lymphoblastic leukemia (ALL) (n = 6, 35.3%), acute myeloid leukemia (AML) (n = 4, 23.5%), primary immunodeficiency (PID) (n = 3, 17.7%), and other hematologic or metabolic disorders (n = 4, 23.5%).

Among the 17 patients, 12 (70.6%) developed acute graft-versus-host disease (aGvHD). All patients received fecal microbiota transplantation (FMT) as a preventive strategy following HSCT. Regarding aGvHD severity, five patients (29.4%) did not develop aGvHD, three (17.7%) developed grade 1 disease, and nine (52.9%) developed grade 2 aGvHD; no cases of grade 3 or 4 were observed. Chronic graft-versus-host disease developed in six patients (35.3%). aGvHD grading was determined based on clinical follow-up over a 6-month period after HSCT. No transplant-related mortality was observed during follow-up. Two patients (11.8%) received antibiotic therapy after FMT (**Table 1**).

**Table 1:** Clinical and demographic characteristics of pediatric HSCT recipients who underwent fecal microbiota transplantation (n = 17).

Characteristics	Value
Age at HSCT, years (median [range])	8.2 (0.7–17.0)
Sex, n (%)	
Male	11 (64.7)
Female	6 (35.3)
Underlying disease, n (%)	
Acute lymphoblastic leukemia (ALL)	6 (35.3)
Acute myeloid leukemia (AML)	4 (23.5)
Primary immunodeficiency (PID)	3 (17.7)
Others	4 (23.5)
aGvHD grade, n (%)	
No aGvHD	5 (29.4)
Grade 1	3 (17.7)
Grade 2	9 (52.9)
Grade 3	0 (0)
Grade 4	0 (0)
Antibiotic exposure after FMT, n (%)	2 (11.8)
Transplant-related mortality, n (%)	0 (0)

**Figure 1:**
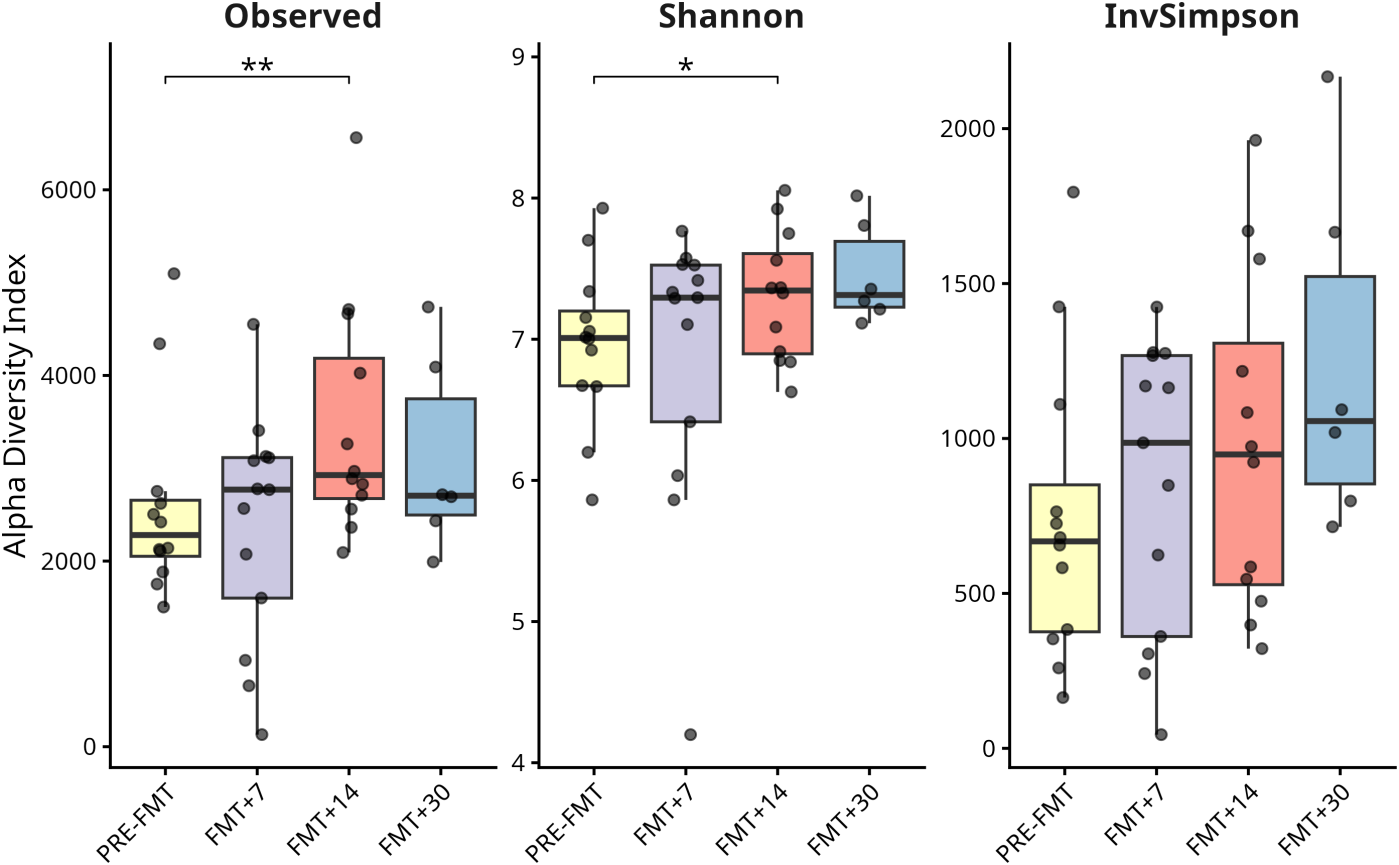
Alpha diversity following FMT. Boxplots show observed richness, Shannon, and inverse Simpson indices at PRE-FMT, FMT + 7, FMT + 14, and FMT + 30. Pairwise comparisons were performed using the Wilcoxon signed-rank test. *p < 0.05; **p < 0.01.

At baseline (PRE-FMT), recipients exhibited reduced alpha diversity, consistent with a disrupted intestinal ecosystem following HSCT. Pairwise comparisons using the Wilcoxon signed-rank test showed a significant increase in observed richness and Shannon diversity at FMT + 14 compared with PRE-FMT, while no significant pairwise differences were detected at FMT + 30, likely reflecting the smaller number of available samples at this later timepoint (**Figure 1**). Nevertheless, alpha-diversity values remained generally higher after FMT, suggesting progressive microbial diversity recovery following intervention. As a complementary longitudinal analysis, linear mixed models using time as a continuous variable demonstrated a significant positive association between days after FMT and inverse Simpson diversity (p = 0.030), while Shannon diversity showed a similar trend (p = 0.062). These findings further support a gradual increase in microbial diversity after FMT (**Supplementary Figure 1**). The number of available fecal samples at each timepoint was as follows: PRE-FMT (n=12), FMT + 7 (n=13), FMT + 14 (n=12), and FMT + 30 (n=6). Some follow-up samples were unavailable because several patients were discharged approximately 20 days after transplantation and continued clinical follow-up in their home provinces, limiting longitudinal stool sample collection. Overall, alpha-diversity analyses revealed the most pronounced changes between days 7 and 14 after FMT, followed by relative stabilization at day 30. Rarefaction curves indicated adequate sequencing depth across samples, with most curves approaching saturation (**Supplementary Figure 2**).

At baseline (PRE-FMT), the gut microbiota of FMT recipients exhibited features consistent with dysbiosis, defined by reduced alpha diversity compared to donors and a compositional imbalance characterized by the dominance of a limited number of genera, including *Lachnoclostridium* (31.1%), *[Ruminococcus] gnavus* group (11.2%), and *Bacteroides* (24.5%). Genera typically associated with intestinal homeostasis, such as *Faecalibacterium* (1.5%), *Akkermansia* (2.6%), and *Blautia* (2.6%), were present at very low relative abundance.

By day 7 post-FMT, marked compositional changes were detected. *Akkermansia* and *Faecalibacterium* increased to 10.4% and 7.1%, respectively, while *Enterococcus* and *Lachnoclostridium* decreased to below 4%.

At day 14, further expansion of *Roseburia* (3.7%), *Blautia* (3.9%), and *Subdoligranulum* (2.1%) was observed, together with continued reductions in *Hungatella* (0.1%) and *[Ruminococcus] gnavus* group (0.7%).

By day 30, the microbial community was characterized by sustained enrichment of *Faecalibacterium* (21.9%), *Blautia* (10.1%), and *Subdoligranulum* (4.2%), along with a marked decline in *Lachnoclostridium* (4.2%) and *Hungatella* (0.3%). *Bacteroides* remained relatively stable, accounting for 22.6% of the community.

**Figure 2:**
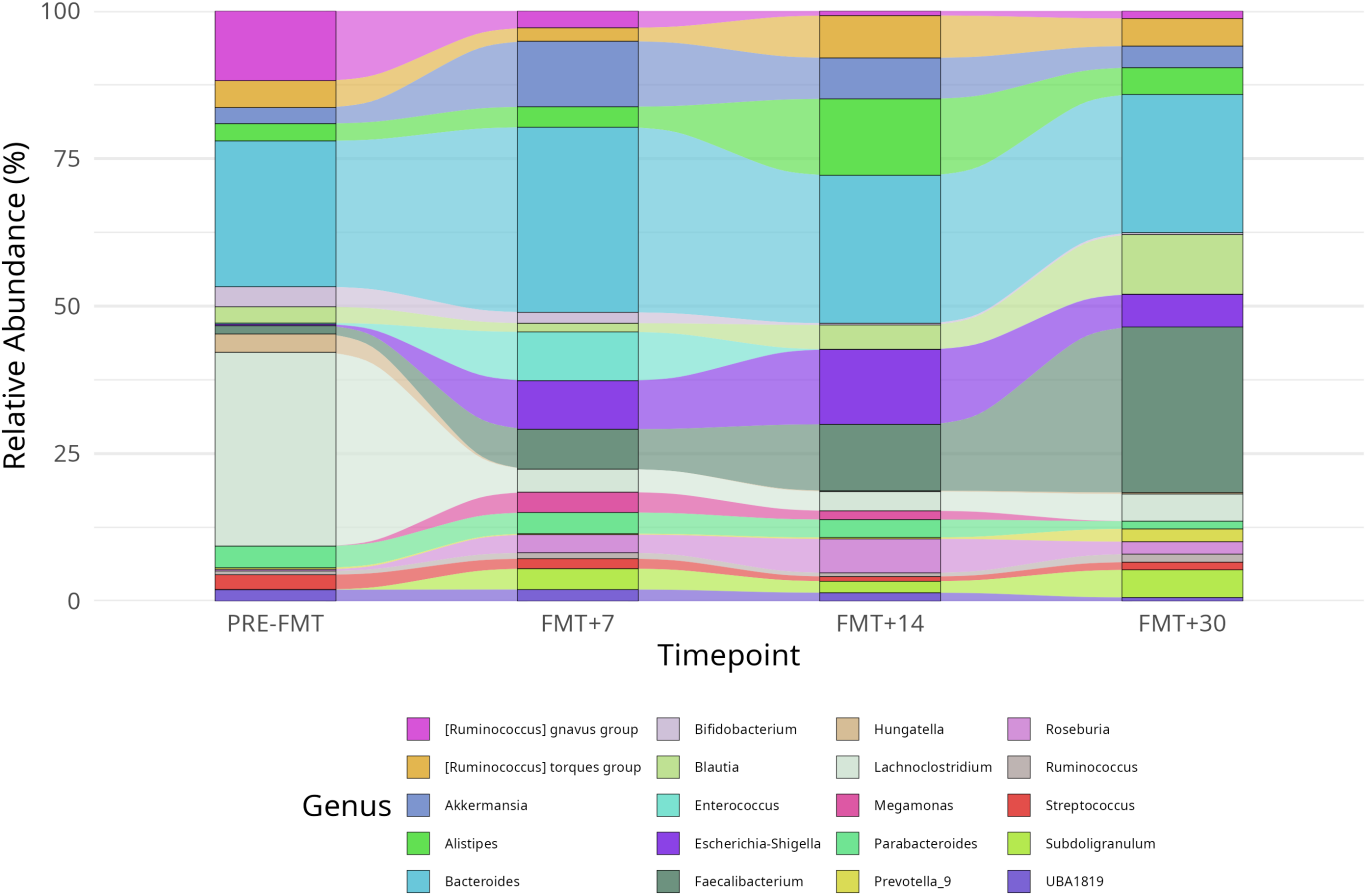
Genus-level relative abundance flow in fecal samples from recipients before and after fecal microbiota transplantation (FMT). The 20 most abundant genera and their temporal dynamics are shown across four time points (PRE-FMT, FMT + 7, FMT + 14, and FMT + 30). Each colored band represents a distinct bacterial genus, and its relative thickness reflects proportional abundance within the microbial community.

Overall, quantitative analysis revealed progressive microbiota reconstruction over time, with depletion of dysbiosis-associated taxa (*Lachnoclostridium*, *Hungatella*) and enrichment of beneficial, short-chain fatty acid–producing genera (*Faecalibacterium*, *Blautia*, *Subdoligranulum*) throughout the 30-day period, as illustrated in **Figure 2**.

### Temporal dynamics of genus-level association networks

Genus-level association networks were constructed at each sampling time point (PRE-FMT, FMT + 7, FMT + 14, and FMT + 30) to explore temporal changes in microbial co-occurrence patterns following fecal microbiota transplantation (FMT) (**Supplementary Figures 3**-**S6**). Prior to FMT (PRE-FMT), the network showed a dysbiosis-associated configuration characterized by limited connectivity and clusters dominated by taxa such as *Lachnoclostridium*, *Hungatella*, *Streptococcus*, and *[Ruminococcus] gnavus* group. Beneficial commensal genera, including *Faecalibacterium* and *Bifidobacterium*, showed limited integration within the network at this time point.

At FMT + 7, the network displayed qualitative changes in association patterns, including the emergence of interactions involving *Faecalibacterium*, *Bacteroides*, and *Parabacteroides*. By FMT + 14, additional associations involving short-chain fatty acid–producing genera such as *Roseburia*, *Subdoligranulum*, *Blautia*, and *Akkermansia* were observed.

At FMT + 30, the network exhibited a broader distribution of associations across multiple genera, including *Blautia*, *Faecalibacterium*, *Bacteroides*, *Alistipes*, and *Akkermansia*. To quantitatively compare network structure across time points, pairwise edge overlap was assessed using the Jaccard index with permutation testing and Benjamini–Hochberg correction. The PRE-FMT and FMT + 30 networks showed significantly lower edge overlap than expected by chance (observed Jaccard = 0.150; permutation p = 0.008; BH-adjusted p = 0.048), indicating a measurable reorganization of genus-level associations by day 30 after FMT.

Overall, these longitudinal analyses suggest progressive restructuring of microbial association patterns over time following FMT, with the largest detectable shift occurring between the PRE-FMT and FMT + 30 states.

### Association between gut microbiota composition and aGvHD organ involvement

**Figure 3:**
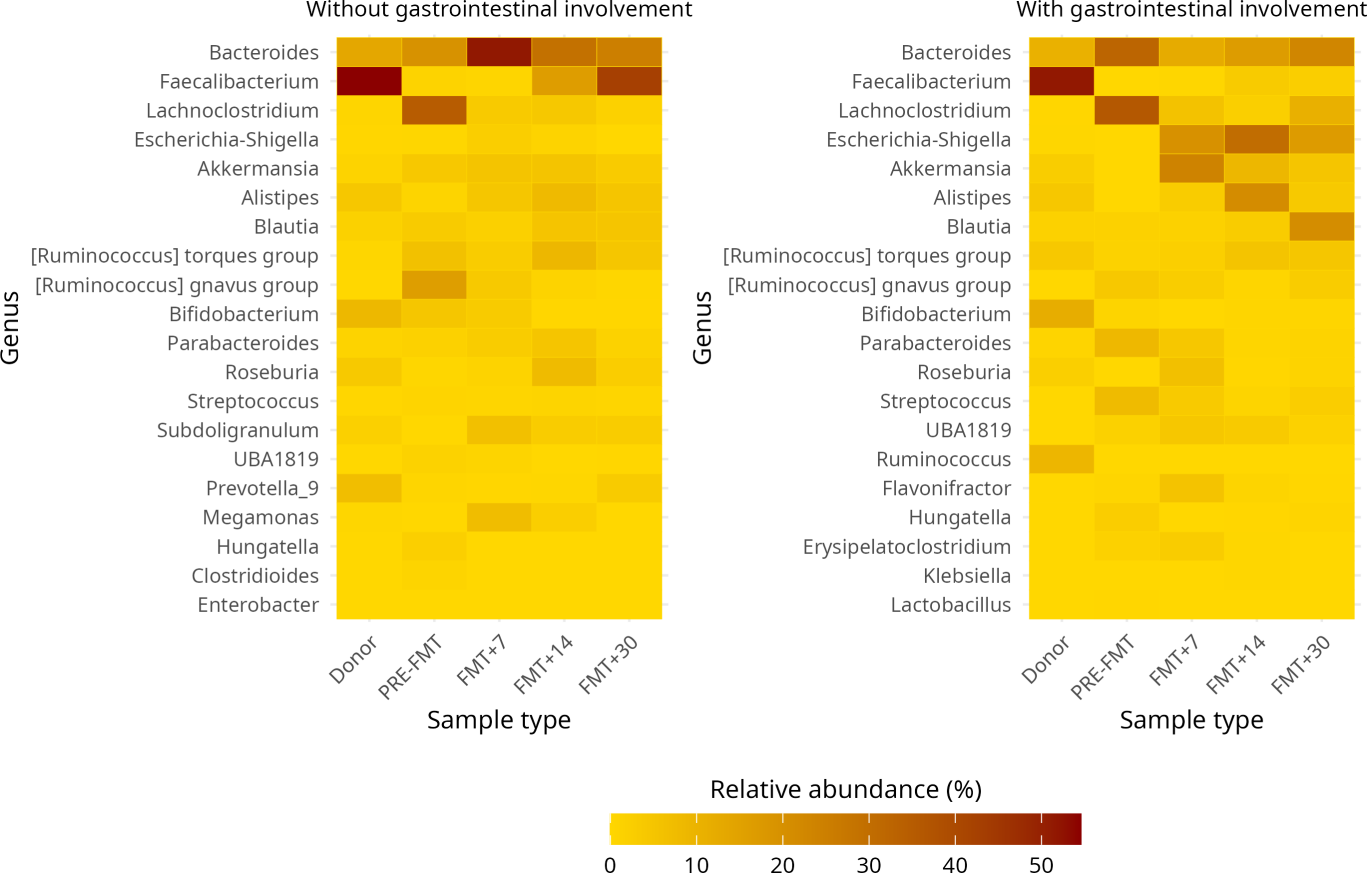
Heatmaps showing the relative abundance of the most prevalent bacterial genera across longitudinal sampling time points in pediatric HSCT recipients. Patients were stratified into two groups: those without aGvHD or with isolated cutaneous involvement (left panel) and those with gastrointestinal involvement (right panel). Columns represent sample types (donor, PRE-FMT, and post-FMT time points), while rows correspond to bacterial genera. Color intensity indicates relative abundance.

To assess whether gut microbial profiles differed according to the pattern of aGvHD organ involvement, we compared the relative abundance of the most prevalent bacterial genera between patients without aGvHD or with exclusively cutaneous involvement (n=8) and those with gastrointestinal involvement (n=9) (**Figure 3**).

Both groups exhibited dynamic compositional changes following FMT; however, distinct microbial configurations were observed. Patients without aGvHD or with isolated cutaneous involvement showed higher relative abundance and temporal persistence of commensal genera such as *Faecalibacterium*, *Bifidobacterium*, and *Lachnoclostridium*, with transient increases at early post-FMT time points. In contrast, samples from patients with gastrointestinal involvement were characterized by a relative depletion of *Faecalibacterium* and enrichment of genera commonly associated with intestinal dysbiosis, including *Escherichia–Shigella*, *Klebsiella*, and *Erysipelatoclostridium*.

The loss of key short-chain fatty acid–producing taxa and the expansion of opportunistic genera in patients with gastrointestinal aGvHD suggest impaired microbial resilience and a more pro-inflammatory gut ecosystem in this subgroup.

## Discussion

This study provides longitudinal evidence of gut microbiota reconstitution in pediatric HSCT recipients following FMT. Our findings showed a progressive restoration of microbial richness and evenness, paralleled by compositional shifts involving enrichment of commensal anaerobic taxa. Early increases in Faecalibacterium, Akkermansia, and Bacteroides, along with the decline of dysbiotic taxa such as Enterococcus and Escherichia–Shigella, is consistent with a transition from a disrupted to a more eubiotic ecosystem. Together, these dynamics support the potential of FMT as a strategy to mitigate HSCT-associated dysbiosis in pediatric patients.

The observed increase in alpha diversity from day 7 onward is consistent with previous studies reporting accelerated microbiota recovery after FMT in immunocompromised hosts (Khanna et al., 2017). The progressive increase in microbial diversity over time suggests recovery of microbial complexity, a feature linked to ecosystem stability and colonization resistance. Restoration of diversity likely reflects ecological restructuring of the gut microbial community following FMT.

At the compositional level, enrichment of Faecalibacterium, Akkermansia, and Roseburia following FMT highlights the re-establishment of short-chain fatty acid (SCFA)-producing bacteria, which are central components of a healthy intestinal ecosystem (Caetano and Castelucci, 2022). The persistence of these genera through day 30 post-FMT suggests sustained compositional changes following the short two-dose regimen used in this study.

Beyond taxonomic composition and diversity, genus-level association network analyses provide a structural perspective on microbiota recovery following FMT. In adult HSCT recipients, a phase II trial demonstrated that early FMT induces extensive reorganization of microbial association networks, reflecting community-level restructuring rather than isolated taxonomic shifts (Rashidi et al., 2023). Consistent with these observations, our network analyses revealed a fragmented pre-FMT configuration with limited connectivity and localized clusters dominated by dysbiosis-associated taxa. Following FMT, progressive reorganization of network topology was observed, characterized by increased connectivity and redistribution of associations across multiple genera. By day 30 post-FMT, the network displayed a broader redistribution of associations across multiple genera. Together, these findings suggest that microbiota recovery after FMT involves coordinated reorganization of microbial association patterns rather than isolated taxonomic changes alone.

Notably, *Blautia*, a member of the Lachnospiraceae family, showed a dynamic pattern, with progressive expansion during the post-FMT period and increased relative abundance by day 30. Although this trend did not reach statistical equivalence with donor levels, *Blautia* has previously been associated with favorable transplant outcomes in allogeneic HSCT recipients, including lower GVHD-related mortality and improved overall survival (Jenq et al., 2015; Biagi et al., 2019). Its increasing representation and integration within post-FMT microbial networks in our cohort may therefore reflect partial restoration of anaerobic microbial communities following FMT.

Due to the high (>99%) sequence similarity between *Escherichia coli* and *Shigella* spp. within the 16S rRNA gene, short-read V3–V4 amplicon sequencing does not allow reliable discrimination between these taxa. Accordingly, sequences were reported jointly as Escherichia–Shigella, following SILVA taxonomy conventions (Větrovský and Baldrian, 2013).

Gut microbiome dynamics after HSCT are influenced by multiple interacting factors, including age, diet, conditioning regimens, immunosuppressive therapies, transplant-related complications, and antibiotic exposure. Therefore, the heterogeneity of this real-world pediatric cohort likely contributed to interindividual variability in microbiome recovery after FMT. Although two patients received systemic antibiotics after FMT, the limited number precluded meaningful subgroup analyses. Pediatric HSCT recipients represent a highly vulnerable immunocompromised population in whom disruption of the intestinal microbiome may favor opportunistic colonization and infectious complications, supporting the importance of integrated microbiological and immunological surveillance strategies during the post-transplant period (Mohammed et al., 2023).

Stratification according to gastrointestinal involvement revealed distinct microbial configurations. Children without gastrointestinal aGvHD involvement showed higher relative abundance of *Faecalibacterium, Bifidobacterium*, and *Lachnoclostridium*, whereas patients with gastrointestinal involvement showed depletion of *Faecalibacterium* and enrichment of *Escherichia–Shigella* and Klebsiella. Notably, several taxa depleted in patients with gastrointestinal involvement are commonly associated with short-chain fatty acid (SCFA) production, particularly butyrate and propionate, which have been associated with intestinal homeostasis through maintenance of epithelial barrier integrity and regulatory T cell–mediated immune tolerance. Consistent with this, adult HSCT studies have linked the loss of SCFA-producing taxa to more severe GVHD phenotypes (Song et al., 2024). However, most evidence connecting gut microbiota features to transplant outcomes derives from adult cohorts, while pediatric data remain limited. Given the developmental plasticity of the pediatric microbiota, our study addresses this gap by providing one of the first longitudinal characterizations of gut microbiota reconstitution following a defined fecal microbiota transplantation intervention in pediatric HSCT recipients.

Although no deaths occurred among the 17 patients analyzed, this study was not powered to assess clinical endpoints such as GVHD reduction or survival, and the absence of a non-FMT control group limits causal inference. The pediatric microbiome in HSCT recipients remains poorly studied, and the currently available literature is still controversial. Therefore, part of the microbiome changes observed in our cohort may reflect the natural recovery process following HSCT. However, previous studies in adult HSCT recipients, including controlled trials, reported differences in microbiome reconstitution between patients who received FMT and those who did not (Rashidi et al., 2023). Accordingly, our primary objective was ecological rather than clinical: to describe temporal patterns of microbial reconstitution following FMT. Controlled pediatric studies integrating metagenomic and metabolomic approaches will be required to link these ecological changes to functional and clinical outcomes.

## Conclusion

Pediatric HSCT recipients represent a unique subgroup, as their gut microbiota is still undergoing developmental maturation, which may influence recovery trajectories following major clinical interventions. In this study, we provide longitudinal evidence that fecal microbiota transplantation promotes progressive restoration of microbial diversity and re-establishment of key short-chain fatty acid–producing taxa, which remain stable during follow-up in pediatric HSCT recipients. These findings suggest that FMT may contribute to stabilization of the intestinal ecosystem after HSCT and support its potential role as a microbiota-based supportive strategy in this vulnerable population.

## Data Availability

The datasets presented in this study can be found in online repositories. The names of the repository/repositories and accession number(s) can be found in the article/**Supplementary Material**.

## Supplementary material

Supplementary Figure 1 Longitudinal analysis of alpha diversity following FMT. Boxplots show observed richness, Shannon, and inverse Simpson indices in donors and recipient samples across timepoints. Time trends were evaluated using linear mixed models with time modeled as a continuous variable. P values for overall temporal trends are indicated in each panel.

Supplementary Figure 2 Rarefaction curves of alpha diversity metrics (Observed richness, Shannon, and inverse Simpson) across sequencing depth for all samples. Curves approaching saturation indicate adequate sequencing depth.

Supplementary Figure 3 Microbial association network at PRE-FMT baseline. Nodes represent bacterial genera, with size proportional to their relative abundance. Edges indicate significant associations (green: positive; red: negative), and edge thickness reflects the strength of these associations. The network depicts microbial interaction patterns at baseline prior to fecal microbiota transplantation (PRE-FMT)

Supplementary Figure 4 Microbial association network at FMT + 7. Nodes represent bacterial genera, with size proportional to their relative abundance. Edges indicate significant associations (green: positive; red: negative), and edge thickness reflects the strength of these associations. The network depicts microbial interaction patterns at 7 days after fecal microbiota transplantation (FMT + 7).

Supplementary Figure 5 Microbial association network at FMT + 14. Nodes represent bacterial genera, with size proportional to their relative abundance. Edges indicate significant associations (green: positive; red: negative), and edge thickness reflects the strength of these associations. The network depicts microbial interaction patterns at 14 days after fecal microbiota transplantation (FMT + 14).

Supplementary Figure 6 Microbial association network at FMT + 30. Nodes represent bacterial genera, with size proportional to their relative abundance. Edges indicate significant associations (green: positive; red: negative), and edge thickness reflects the strength of these associations. The network depicts microbial interaction patterns at 30 days after fecal microbiota transplantation (FMT + 30).
