# Correction: Fecal microbiota transplantation promotes gut microbiome recovery in pediatric hematopoietic stem cell transplant recipients

**DOI:** 10.3389/frmbi.2026.1904862

**Published:** 2026-06-17

**Authors:** María Florencia Fernandez, Abigail Stricker, Adriana Bottero, Laura Busquet, Carlos Waldbaum, Fabiana López Mingorance, Raúl Martinez Patetta, Ignacio Toer, Ana Juliá, Andrea Mangano

**Affiliations:** 1 Department of Microbiology, Hospital de Pediatría “Prof. Dr. Juan P. Garrahan”, Buenos Aires, Argentina; 2 Department of Gastroenterology, Hospital de Pediatría “Prof. Dr. Juan P. Garrahan”, Buenos Aires, Argentina; 3 Division of Gastroenterology, Hospital de Clínicas “José de San Martín”, Buenos Aires, Argentina; 4 Bone Marrow Transplant Unit, Hospital de Pediatría “Prof. Dr. Juan P. Garrahan”, Buenos Aires, Argentina

**Keywords:** 16S rRNA sequencing, acute graft-versus-host disease, dysbiosis, fecal microbiota transplantation, gut microbiome, microbiome diversity, pediatric hematopoietic stem cell transplantation

The publication of this article was supported by “Fundación Garrahan”, who helped cover the article processing charges (APCs). The **Funding** statement has been updated as follows:

“The author(s) declared that financial support was received for this work and/or its publication. This work was partially supported by Fundación Bunge y Born, by Fundación Garrahan, and by Fondo Sectorial Argentino, Ministerio de Ciencia, Tecnología e Innovación (Argentina) (Grant PICTO-2021-UCTH, No. 0004)”.

The original version of this article has been updated.

