# Supplementary figures and images for: Fecal microbiota transplantation promotes gut microbiome recovery in pediatric hematopoietic stem cell transplant recipients
Source: Front Microbiomes. 2026 Jun 5;5:1849762. doi: 10.3389/frmbi.2026.1849762 (PMC13322138; doi:10.3389/frmbi.2026.1849762)

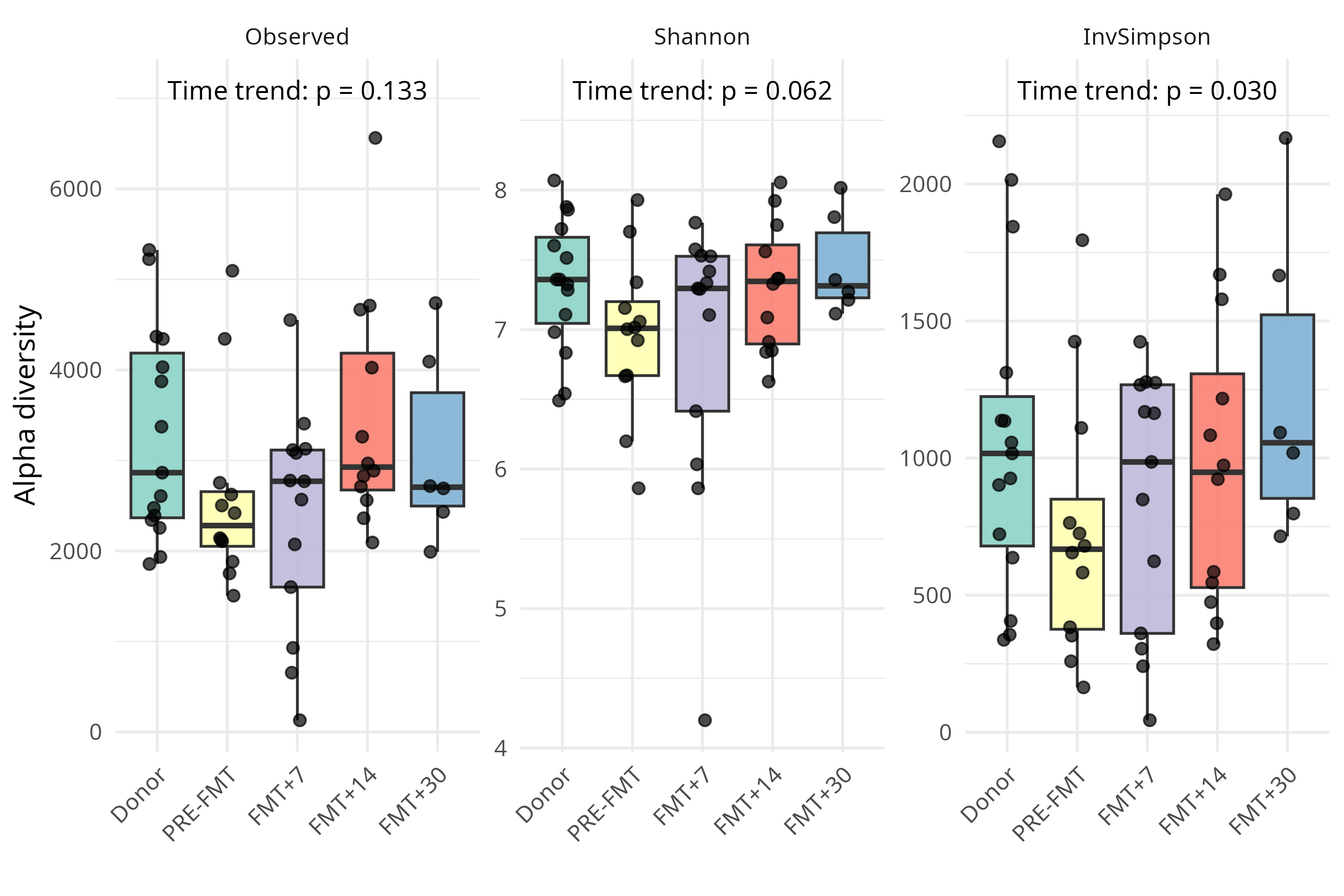

Supplement: Supplementary Figure 1 — Longitudinal analysis of alpha diversity following FMT. Boxplots show observed richness, Shannon, and inverse Simpson indices in donors and recipient samples across timepoints. Time trends were evaluated using linear mixed models with time modeled as a continuous variable. P values for overall temporal trends are indicated in each panel. [file Image1.tiff]

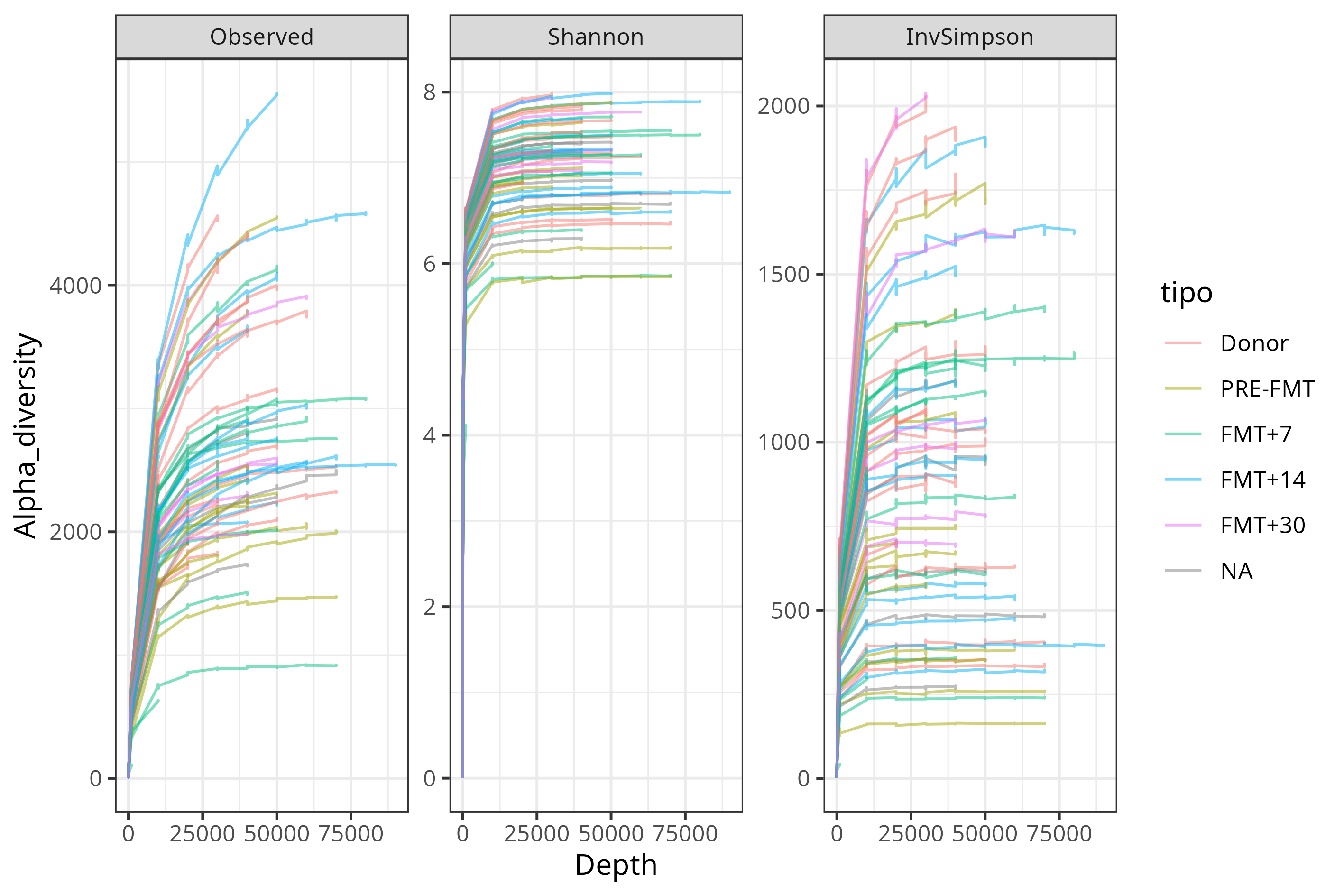

Supplement: Supplementary Figure 2 — Rarefaction curves of alpha diversity metrics (Observed richness, Shannon, and inverse Simpson) across sequencing depth for all samples. Curves approaching saturation indicate adequate sequencing depth. [file Image2.tiff]

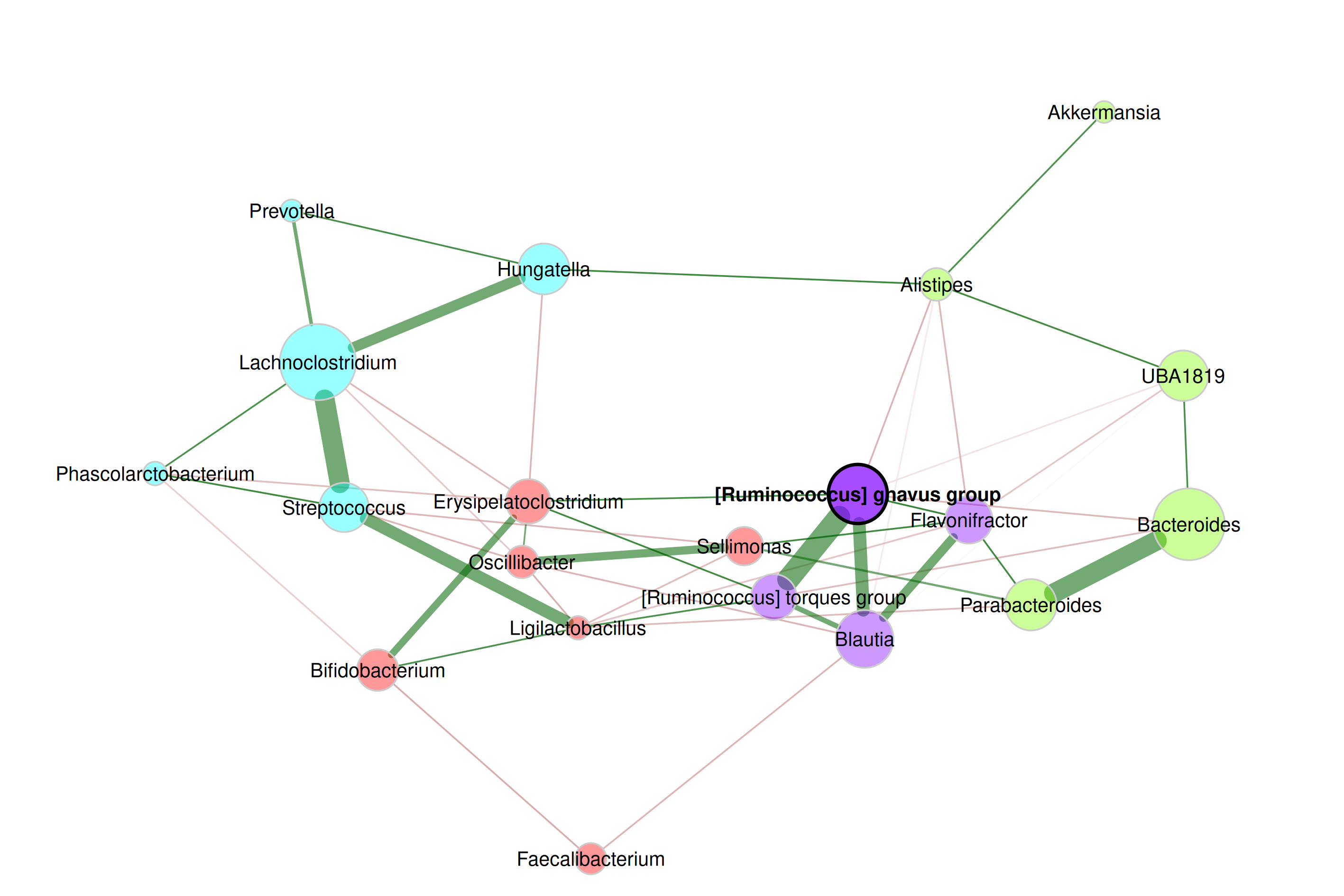

Supplement: Supplementary Figure 3 — Microbial association network at PRE-FMT baseline. Nodes represent bacterial genera, with size proportional to their relative abundance. Edges indicate significant associations (green: positive; red: negative), and edge thickness reflects the strength of these associations. The network depicts microbial interaction patterns at baseline prior to fecal microbiota transplantation (PRE-FMT) [file Image3.tiff]

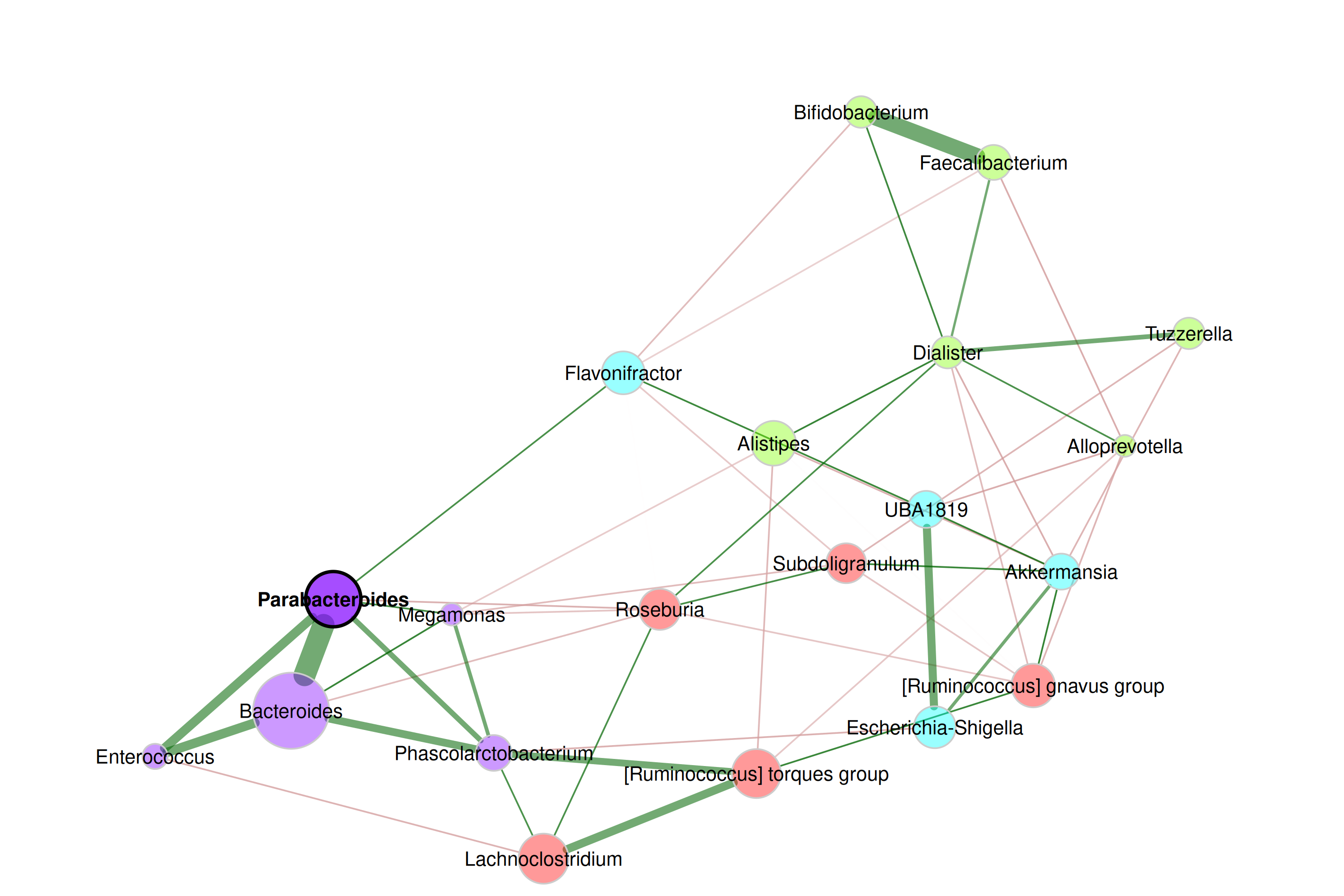

Supplement: Supplementary Figure 4 — Microbial association network at FMT + 7. Nodes represent bacterial genera, with size proportional to their relative abundance. Edges indicate significant associations (green: positive; red: negative), and edge thickness reflects the strength of these associations. The network depicts microbial interaction patterns at 7 days after fecal microbiota transplantation (FMT + 7). [file Image4.tiff]

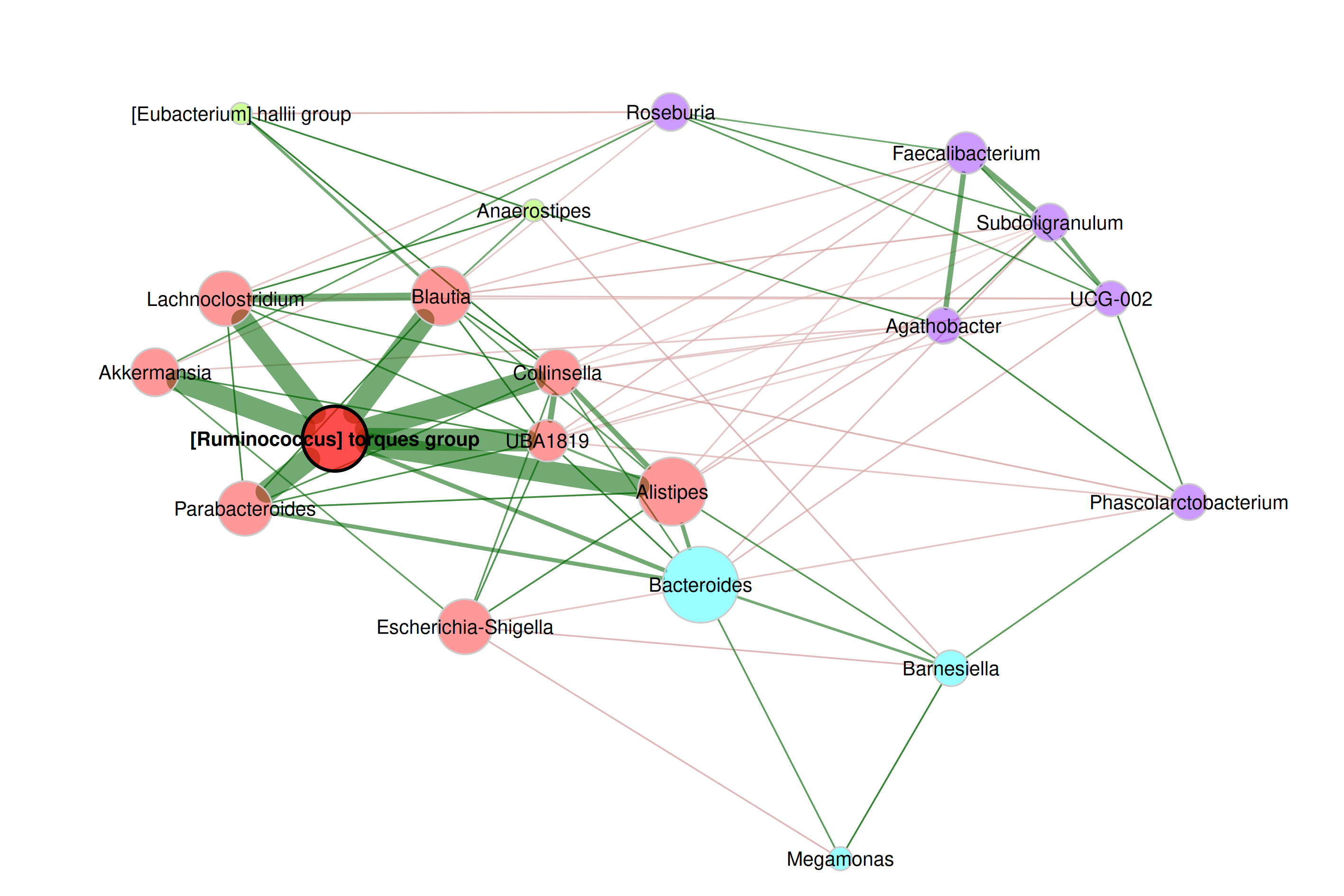

Supplement: Supplementary Figure 5 — Microbial association network at FMT + 14. Nodes represent bacterial genera, with size proportional to their relative abundance. Edges indicate significant associations (green: positive; red: negative), and edge thickness reflects the strength of these associations. The network depicts microbial interaction patterns at 14 days after fecal microbiota transplantation (FMT + 14). [file Image5.tiff]

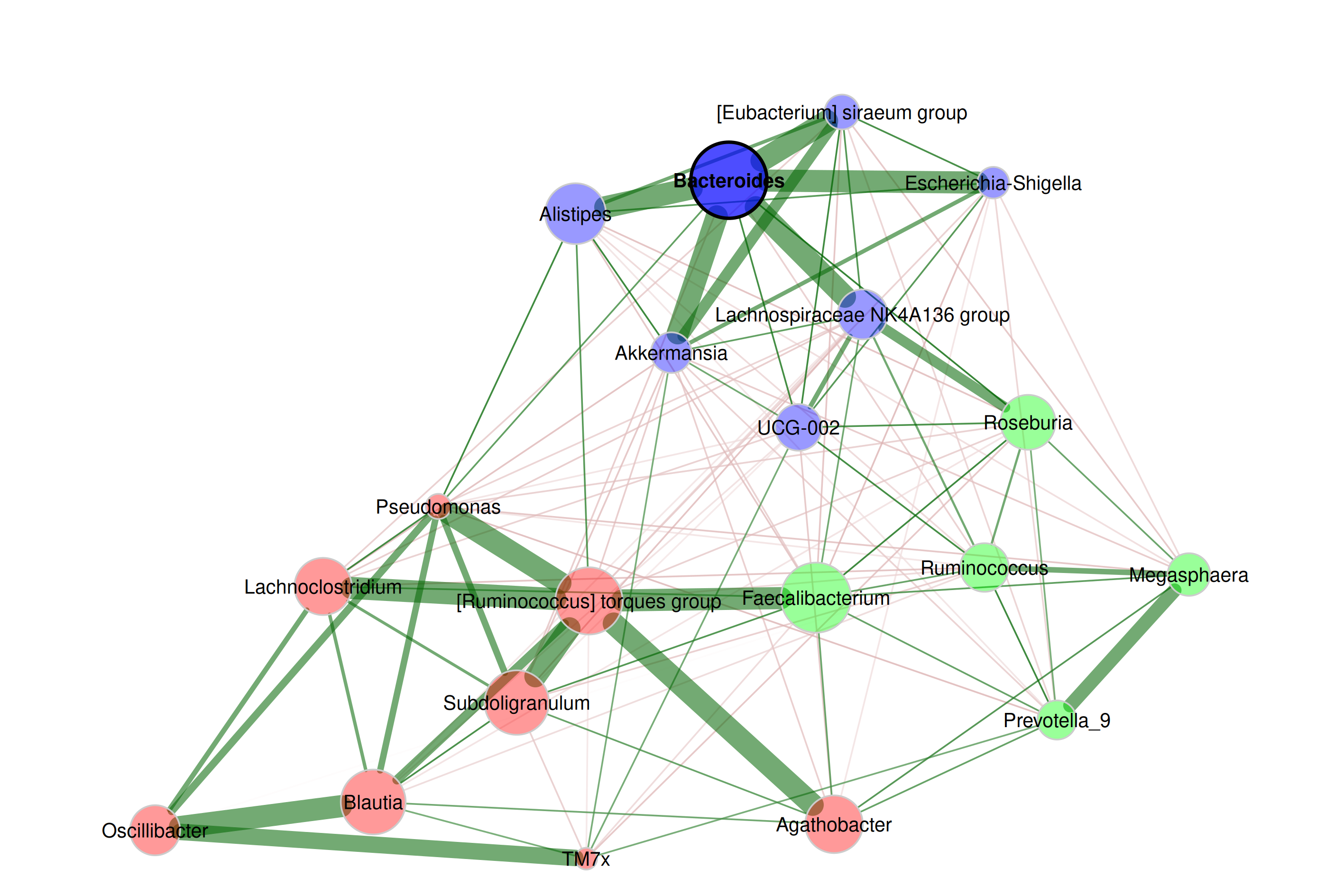

Supplement: Supplementary Figure 6 — Microbial association network at FMT + 30. Nodes represent bacterial genera, with size proportional to their relative abundance. Edges indicate significant associations (green: positive; red: negative), and edge thickness reflects the strength of these associations. The network depicts microbial interaction patterns at 30 days after fecal microbiota transplantation (FMT + 30). [file Image6.tiff]
